# Foreign Bodies in the External Auditory Meatus

**Published:** 1907-06-29

**Authors:** 


### OTOLOGY.

### FOREIGN BODIES IN THE EXTERNAL AUDITORY MEATUS.

These are of three main classes—animate and inanimate—and, of the latter, organic and inorganic. Flies, cockroaches, and other creeping things are occasionally found in the external auditory meatus. The bitterness of the ceruminous secretion no doubt accounts for the infrequent visits of insects of this class. When found alive they should be killed with a few drops of perchloride of mercury lotion; 1—4,000 is a suitable strength. They can then usually be dislodged with the syringe. To the class of inanimate organic bodies orange pips, peas, rice, and cherry stones belong. Some of these absorb moisture and swell in consequence; they thus press on the meatal walls, and are difficult to dislodge with the syringe. A snare formed with a loop of fishing gut, horsehair, or fine wire may secure a hold of these objects. Forceps are not to be recommended, except in specially skilled hands. Of the inorganic foreign bodies, beads, pebbles, shells, india-rubber, and the like are sometimes met with. As they do not absorb moisture and so swell up, they are much less likely to become impacted; they should be removed by syringing. An inspissated black collection of cerumen should be readily recognised, and not mistaken for a foreign body. To remove cerumen, if the simple method of syringing fail, the mass should be first disintegrated by the use of warm alkaline drops (sodium bicarbonate, 10 grains, in water, to 1 oz.). When softened by this mode of treatment cerumen is easily syringed out.

The rule is to syringe along the roof of the meatus, While doing so the meatal canal should be straightened, by gentle traction of the pinna backwards and a little upwards. If, however, careful inspection reveals a narrow cleft between some other part of the meatal wall and the object to be dislodged, then into this cleft the fluid should be directed.

Parts of the meatal walls are exceedingly sensitive, and the lining cutis is easily abraded with instruments. The floor and posterior walls are not quite so sensitive as the anterior and antero-superior walls. Pain is produced when the tympanic membrane is touched, especially in the upper part. A knowledge of the less acutely sensitive areas of the meatus and of the membrane enables one to dislodge a foreign body with a snare or blunt rectangular hook, without danger and without anæsthesia in many cases.

The most difficult foreign bodies to remove are those so large that they become engaged half-way down the canal at the junction of the cartilaginous with the osseous meatus. They are especially difficult if attempts to remove them with forceps having failed, have resulted in abrasion of the meatal walls, which consequently become excessively tender and swollen. The difficulty is increased if the foreign body is of the class which absorbs moisture. Not uncommonly, attempts to dislodge such impacted objects have led to their propulsion to the deeper parts, where the canal is frequently somewhat more roomy. Here they may rest against the tympanic membrane, and by pressing on the upper half of the processus brevis and the handle of the malleus causes considerable pain and tinnitus aurium.

If resting on the lower half of the membrane in the sinus of the meatus, the foreign body will cause discomfort and tinnitus, but not necessarily actual pain.

In these cases a general anæsthetic may be necessary, and the meatus should be opened with strict antiseptic precaution through a post-aural incision. With the use of a blunt hook and prehensile forceps, under guidance of a good head light, the foreign body can be safely removed, and the sutured wound will be expected to heal by first intention.

